# Small Bowel Obstruction due to Non-steroidal Anti-inflammatory Drug-induced Diaphragm Disease: A Case Report

**DOI:** 10.7759/cureus.2350

**Published:** 2018-03-20

**Authors:** Hang-Fai So, Ian Bloomfield

**Affiliations:** 1 General Surgery, Logan Hospital

**Keywords:** diaphragm disease, small bowel obstruction

## Abstract

Small bowel obstruction (SBO) is a common illness encountered by general surgeons. However, obstruction caused by diaphragm disease induced by non-steroidal anti-inflammatory drug (NSAID) is exceedingly rare. The diagnosis is challenging as the signs and symptoms are neither sensitive nor specific. We report the case of a 59-year-old male who presented with SBO secondary to this uncommon condition. We hope to raise awareness of this unusual entity.

## Introduction

Small bowel obstruction (SBO) is a common condition treated by general surgeons. It can be precipitated by a myriad of factors, broadly divided into extrinsic, intramural, or intraluminal conditions. SBO secondary to diaphragm disease induced by non-steroidal anti-inflammatory drugs (NSAID) is exceptionally rare. We discuss the case of a 59-year-old male who presented with this uncommon entity.

## Case presentation

A 59-year-old male presented to the emergency department with a three-week history of worsening colicky central and lower abdominal pain. The pain was exacerbated by food. His bowels had been opening regularly in small amounts with the aid of laxatives. He had a 2 kg weight loss over the last few months. He had osteoarthritis of the knee, for which he had started using meloxicam 15mg daily for the last nine months. He was otherwise generally fit with no other medical or surgical history.

On examination, he was afebrile and hemodynamically stable. His abdomen was tender in the right lower quadrant and peri-umbilical region; there were no signs of peritonism.

His blood tests showed C-reactive protein 9.6, white cell count 9.6 x 10^9^/L, haemoglobin 128 g/L, liver functions and renal functions were all within normal limits.

**Figure 1:**
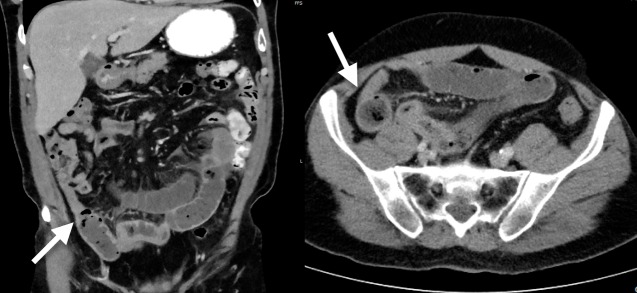
CT scan of the abdomen showing SBO and transition point (white arrow) CT: computed tomography; SBO: small bowel obstruction.

He proceeded to have a computed tomography (CT) scan of the abdomen. This showed a partial SBO with dilatation of the mid to distal small bowel and faecalisation of intraluminal material in the distal segments. There was a transition point in the right iliac fossa with a partial collapse of the distal small bowel (Figure 1).

He had a trial of 24 hours of conservative management, which included clear fluid diet, analgesia, and anti-emetics. His symptoms worsened so he proceeded to have a diagnostic laparoscopy on day two of admission. At laparoscopy, he had a copious amount of serous free fluid in the abdomen and dilated small bowel with evidence of congestion. However, no clear cause of bowel obstruction was identified. The decision was made to convert to mini-laparotomy to deliver the small bowel and palpate for any intrinsic lesions. Two concentric strictures were felt in the distal jejunum and these were observed to be the cause of obstruction. The strictured small bowel was resected and an end to end hand-sewn anastomosis was performed. The mesenteric defect was closed and his abdominal fascia and skin were closed. 

He had a non-complicated recovery in the ward. His diet was progressively upgraded and his bowels were opened on day three post operation. He was discharged from hospital on day four after tolerating a full diet. A follow-up phone call three weeks post operation confirmed that he had made a full recovery with no further complaints.

**Figure 2:**
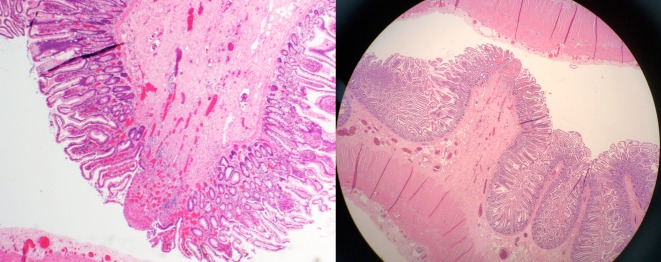
Histology showing features of diaphragm disease

Macroscopic examination of the bowel specimen revealed a narrowed band on the serosal surface of the bowel and the underlying bowel wall felt thickened. Microscopically, there were occasional foci of mucosal ulceration which appeared to be located at the tip of folds within the small intestine. Some of these foci of ulceration were associated with granulation tissue within the lamina propria. Foci of ulceration were associated with focal crypt dropout, focal cryptitis, and crypt abscess formation. The adjacent epithelium focally showed reactive appearing atypia and occasional intraepithelial neutrophils. Immediately deep to some of these areas of ulceration, there appeared to be increased fibrosis of the submucosa. Whilst no classical diaphragms were identified macroscopically, the microscopic features were consistent with NSAID-induced diaphragm disease (Figure 2).

## Discussion

SBO is a common problem that has many different causes. In the context of this case, stricturing small bowel disease in a virgin abdomen can be due to a congenital band, inflammatory bowel disease, small bowel malignancy such as lymphoma, enteropathy secondary to ischemia, or in this case, diaphragm disease.

First formally described by pathologist Lang in 1988 [1], diaphragm disease is a rare condition of the gastrointestinal mucosa due to prolonged NSAID use. A systemic review reported only 55 cases of small bowel diaphragm disease requiring surgery over a 30-year period [2]. The pathogenesis of this illness is not well understood, cyclo-oxygenase and prostaglandin inhibition have been implicated to cause injury to enterocytes and villous microcirculation, and the subsequent inflammatory reaction leads to ulceration. This ulceration then develops circumferential fibrosis, resulting in the formation of a stricture resembling the characteristic “diaphragm” [3]. These are located most commonly in the ileum, causing multiple stenoses of the lumen [4]. It has been reported that 40%–70% of the patients on long-term NSAID develop small bowel enteropathy. However, the incidence of NSAID-induced diaphragm disease is only around 2% [5]. The low incidence is probably because many cases remain subclinical, with only a handful of cases presenting with SBO. Diaphragm disease in which NSAID intake could not be proven has also been published [6].

Diagnosis based on history and clinical findings alone is difficult, as the symptoms and signs are usually non-specific. This is demonstrated by our patient, who presented with non-specific intermittent abdominal pain and weight loss. Imaging by CT also has limited value in confirming this diagnosis, as diaphragms can appear as exaggerated plica ciculares and they are either not seen or easily interpreted on CT [7]. Capsule endoscopy is able to diagnose this entity but has the risk of the capsule being incarcerated in the diaphragm, thus causing SBO and precipitating an immediate surgical emergency [8].

Even at laparoscopy, findings often do not point to this specific diagnosis. The bland serosal appearance of the diaphragm often escapes the watchful eye of the surgeon. Usually, the diseased segment of the small bowel is only found by meticulous palpation of the entire length of the bowel during laparotomy.

Management of SBO due to diaphragm disease involves surgical treatment and cessation of NSAID. At surgery, both stricturoplasty and resection have been reported with success. The general preference is resection because the diagnosis is often not clear at surgery [2]. Surgical treatment of diaphragm disease is potentially curative.

## Conclusions

Since SBO caused by diaphragm disease is rare, its diagnosis requires a high index of suspicion. This case highlights the importance of thorough history taking and meticulous attention to details during surgery in order to bring about a favourable outcome to this rare disease.
